# Understanding University Students’ Willingness to Try Cultured Meat: Insights from Italy

**DOI:** 10.3390/nu18152418

**Published:** 2026-07-24

**Authors:** Azzurra Annunziata, Artur Kraus, Angela Mariani

**Affiliations:** 1 Department of Economics, Law, Cybersecurity, and Sports Science, University of Naples Parthenope, 80132 Naples, Italy; 2 Department of Economics and Finance, University of Rzeszow, 35-601 Rzeszow, Poland; akraus@ur.edu.pl; 3 Department of Economic and Legal Studies, University of Naples Parthenope, 80132 Naples, Italy; angela.mariani@uniparthenope.it

**Keywords:** cultured meat, consumer acceptance, willingness to try, logistic regression, neophobia, acceptance alternative proteins, food technophobia

## Abstract

**Background:** Cultured meat (CM) has emerged as a promising, albeit controversial, alternative to conventional livestock production, offering potential benefits in terms of sustainability, animal welfare, and resource efficiency. Despite these potential advantages, consumer acceptance remains uncertain, particularly in countries where CM is not yet commercially available. **Objectives**: this study investigates the determinants of willingness to try (WTT) cultured meat among Italian university students (n = 335), with particular attention to the role of product-related perceptions and personal values and motivations. **Methods**: data were collected through an online survey and analyzed using binary logistic regression to identify the main drivers of respondents’ willingness to try CM. **Results**: the findings suggest that acceptance of CM among university students is driven primarily by familiarity, perceived safety, and ethical considerations, particularly those related to animal welfare, rather than by demographic characteristics or resistance to novel food technologies. **Conclusions**: these findings offer practical implications for policymakers and industry stakeholders, highlighting the importance of transparent communication strategies emphasizing product safety and animal welfare benefits as a means of increasing familiarity with and acceptance of CM among younger consumers.

## 1. Introduction

Over the past decades, global demand for meat has increased significantly, mainly due to population growth and rising incomes [1]. This growth in demand is associated with significant environmental, ethical, and public health concerns. Conventional livestock production represents one of the most significant sources of environmental pressure within global agrifood systems, accounting for a significant share of greenhouse gas emissions [2]. Livestock production is also a major driver of deforestation, biodiversity loss, and marine eutrophication, with impacts strongly influenced by feed production systems and international feed trade [3]. Furthermore, it has been pointed out that crops used for animal feed can reduce the overall efficiency of the food system and affect food security [4]. Intensive animal farming is also associated with ethical concerns regarding animal welfare and contributes to public health risks, including zoonotic disease transmission and antimicrobial resistance [5].

In response to these challenges, a growing body of literature suggests, on the one hand, strategies and measures to improve the sustainability of livestock farming [6,7], and, on the other hand, a transition aimed at reducing meat overconsumption and replacing animal proteins with alternative sources [8,9]. In this context, cultured meat (CM)—animal cells grown in vitro for consumption—has emerged as one of the most recent and controversial food technologies, proposed as a more sustainable alternative to conventional meat production, with potential benefits in terms of environmental sustainability, animal welfare, human health, and resource efficiency.

As a new food production technology still in its early stages, CM is the subject of widespread debate about its advantages and disadvantages, with production costs still substantially higher than those of conventional meat [10] and conflicting views emerging between promising narratives and counter-narratives [11]. Regarding environmental impacts, findings suggest that, although CM could substantially reduce land and water use, as well as greenhouse gas emissions, its considerable energy requirements due, among other factors, to the electricity needed for bioreactors and its reliance on hypothetical laboratory-scale production data constrain the reliability of current environmental assessments [12,13]. One strand of the literature highlights that CM has the potential to enhance food safety through controlled production environments and to provide health benefits via optimized nutritional profiles. For instance, CM could be enriched with health-beneficial fatty acids, such as omega-3, while containing lower levels of saturated fats [12,14,15]. However, the FAO and WHO (2023) report identifies several potential hazards, with varying probabilities and degrees of severity, that are unique to cell-based food production, including contamination in nutrient-rich culture media and potential chemical hazards from inputs and processing aids [16].

In addition, CM market entry is subject to regulatory approval, which varies considerably across jurisdictions worldwide due to differing regulatory approaches and precautionary frameworks [16]. To date, only a limited number of countries—namely Singapore, the United States, Israel, Australia, and New Zealand—have authorized the commercial sale of specific CM products for human consumption [10,17]. Within the European Union, CM falls under the scope of the Novel Food Regulation (EU) 2015/2283, which requires pre-market safety assessment and authorization [18]. Some EU Member States have expressed reluctance to approve CM. In particular, Austria, France, and Italy have argued that “artificial” food production methods based on cultivated cells may threaten the traditional European agricultural model, have called for stricter evaluation criteria by EFSA, and have raised concerns regarding product nomenclature to ensure that consumers are not misled. Italy has recently attracted international attention as the first country to promulgate a ban on the production and commercialization of food products derived from cell-cultured techniques (national law n. 172/2023). This decision has contributed to making CM a highly polarized issue in the country [19] and has been criticized for conflicting with EU procedures [20].

As with any novel food, the success of CM is contingent on consumer acceptance and willingness to try it [21]. Given that CM is not yet commercially available on a large scale, it is challenging to obtain real data on consumer willingness to try it [22]. Recent research indicates that consumers’ acceptance of CM is far from guaranteed and underlines the need to further explore the determinants of acceptance and the main barriers [10,23,24]. Despite an increasing number of studies on this topic in recent years, whether consumers will accept CM remains unclear [25,26], and the existing evidence remains predominantly concentrated in a limited number of countries [23]. With reference to Italy, the existing literature reports mixed findings. Although some recent studies have reported relatively high acceptance of CM among Italian consumers [19,24], other research has identified lower acceptance levels [27] or lower willingness to consume CM regularly compared with that of consumers from other countries [28]. These inconsistent findings suggest that further evidence is needed.

Based on this background, the current study examines Italian university students’ willingness to try (WTT) CM from the perspective of the Diffusion of Innovations theory (DOI) proposed by Rogers (2003) [29].

The paper focuses on university students as young consumers who are expected to represent the primary target market in the future for CM [30,31] and are widely recognized as potential early adopters of novel food products [31]. Moreover, they tend to exhibit higher levels of pro-environmental behavior and are frequently regarded as pioneers of market change, making them a promising target group for sustainable and innovative food products [32]. Although Italy has one of the oldest populations in Europe, with younger age groups accounting for a relatively small share of the population, previous market research has suggested that these younger consumers may represent the most promising target market for CM [33,34]. Furthermore, from a managerial perspective, reaching consumers at this stage of life may facilitate the development of long-term relationships with the market, as food-related behaviors and preferences formed during early adulthood are likely to persist over time [35].

The added value of this article is twofold: first, it is grounded in DOI theory, a framework with promising applications in the study of CM adoption that remains relatively underexplored in the existing literature [25]; second, it was conducted in a Mediterranean country which, despite the increase in published research over the past year, e.g., [19,24,28], is still underrepresented in the literature [36,37]. Hence, this manuscript offers an incremental and context-specific contribution to the existing literature by providing evidence from a sample of Italian university students within the DOI framework.

## 2. Theoretical Background and Literature Overview

Although many consumers express curiosity towards CM, the literature underscores that their willingness to consume it still faces significant barriers associated with multiple and complex factors [21,37,38].

As a novel food produced using unconventional food technology, CM requires consumers to evaluate an unfamiliar product under conditions of considerable uncertainty. From this perspective, a useful theoretical framework to refer to is the Diffusion of Innovation theory (DOI) developed by Rogers (2003) [29]. According to this theory, the adoption of an innovation is influenced by how potential adopters perceive its characteristics and by the extent to which uncertainty is reduced through communication and information exchange [29]. Specifically, the DOI framework identifies five key attributes that shape the rate of adoption: relative advantage (the perceived superiority of an innovation over existing alternatives), compatibility (the consistency with users’ values, beliefs, and lifestyle), complexity (the perceived difficulty of understanding and using an innovation), trialability (the degree to which an innovation can be experimented before adoption), and observability (the extent to which its results or benefits are visible and can be easily communicated to others) [25,29].

The DOI framework has also proven valuable in explaining and predicting the diffusion of food-related innovations [39] in the context of alternative proteins and CM [25,40].

With reference to *perceived relative advantage,* consumers may perceive CM advantages in terms of both product-related attributes [41,42,43] and broader potential social and environmental benefits [21]. The former include sensory expectations, particularly taste and texture, which have been shown to significantly influence WTT [41,44]. Consumers often expect that CM to have an inferior taste or, at least, a taste different from conventional meat [45], and this perception significantly reduces their WTT, particularly among younger consumers and regular meat eaters [38]. Price also represents a significant barrier, as consumers are generally unwilling to pay a premium for CM compared to conventional meat, and this willingness decreases as the price increases [46,47,48,49]. Among its potential benefits, concerns about the condition of animal welfare and environmental sustainability have been found to positively influence both willingness to adopt CM and willingness to pay [34,50,51], and they have been shown to partially counterbalance neophobic tendencies [41,52].

As for *compatibility*, CM may conflict with consumers’ beliefs about the naturalness and authenticity of food [25]. Perceived unnaturalness has consistently emerged as a major obstacle, often eliciting feelings of disgust that strongly predict rejection [53,54]. At the same time, individual personal psychological traits, such as food neophobia and food technology neophobia, are frequently identified as barriers to the consumption of novel protein sources, including CM [19,27,55]. However, the influence of these factors is not consistent across studies [56]. While some research reports a strong negative relationship [42,53], other studies suggest that their effects may be weaker or even non-significant, particularly among younger consumers [27,57].

In terms of *complexity*, previous research indicates that greater familiarity with and knowledge of CM are generally associated with a higher level of acceptance [21,58]. Commonly, CM is perceived to be difficult to understand by consumers, raising concerns regarding food safety and possible long-term health effects [25,27]. Perceived safety and potential health risks have been identified as stronger determinants of WTT towards CM [23,34,59]. Conversely, prior knowledge and a more comprehensive understanding of the CM production process have been shown to reduce risk perception [24,34,38].

Given that CM is not yet commercially available on a large scale, its *trialability* is limited. However, prior research suggested that exposure to information about this new product, together with the opportunity to try it, may improve attitudes and foster purchase intentions [60]. Rolland et al. [61] found that actual tasting experiences tend to improve acceptance, suggesting that negative expectations are often anticipatory rather than based on direct experience.

With reference to *observability,* the way in which CM is presented through communication strategies strongly influences consumer perceptions [43,62]. In this regard, the use of terminology such as “laboratory” or “in vitro” has been observed to evoke adverse associations among consumers [23,62]. More broadly, the way information is framed and disseminated influences consumers’ perception of risk [30,63]. For instance, former studies have shown that framing CM within familiar food acquisition, preparation, and consumption practices is more effective than emphasizing its production process or presenting it primarily as a novel food product, thereby drawing attention to its technological novelty [64].

From the perspective of DOI, socio-demographic variables may be less important than individuals’ perceptions of the innovation itself. Conversely, the existing literature on CM acceptance also highlights the relevance of these variables, although findings remain mixed [5,52,56,65]. Some studies report higher acceptance among men [24,66,67], whereas others find greater acceptance among women [41,47]. Similarly, although younger individuals are often found to be more open to CM [28,34], evidence from other contexts suggests that older consumers may also show high levels of support [49]. Regarding education, most studies have reported a positive association between educational level and attitudes towards CM [50,67], whereas others have found that education is not significantly related to CM acceptance [30].

Against this background, the present study aims to provide a better understanding of the mechanisms underlying the early diffusion of CM in a pre-market context by investigating (*a*) whether the intention to try CM is primarily influenced by product-related attributes or by personal value motivations and (*b*) how these drivers relate to the different attributes proposed by the DOI framework.

## 3. Materials and Methods

### 3.1. Questionnaire

A structured online survey was designed to collect primary data from university students enrolled at a large university in southern Italy. As CM has not yet been authorized for commercialization in Italy, participants were asked in the introductory section to imagine a hypothetical scenario in which CM was commercially available and to answer the questionnaire accordingly.

To avoid confusion or misperception about the subject of the survey, and in line with previous research [30,67], participants were provided at the beginning of the questionnaire with a definition of CM together with a graphic description. The following base definition of CM was presented: “Cultured meat (also known as cultivated, lab-grown, or cell-based meat) is real animal protein produced by in vitro cell culture techniques, rather than by raising and slaughtering animals. It is created by taking animal stem cells, nourishing them with nutrients, and growing them in bioreactors to form muscle and fat tissues, making it biologically identical to conventional meat”.

The questionnaire included both questions and multi-item psycho-attitudinal scales validated in previous studies on CM and, more generally, on alternative proteins. In the first part of the survey, participants were asked about their meat-eating habits, their willingness to reduce meat consumption, and their concerns about animal welfare when making daily food choices. Subsequently, familiarity with and knowledge of CM were assessed. Familiarity (subjective) was assessed both in general, with respect to alternative protein sources, with the question “How familiar are you with innovative protein sources alternative to animal meat?”, and specifically with respect to CM, with the question “Have you ever heard about cultured meat?”, using a 7-point Likert scale (1 = I’ve never heard of it; 7 = I know it very well) [67,68]. Objective knowledge was assessed using multiple-choice questions in order to assess participants’ knowledge of the product and its production process, as well as their awareness of the regulatory status and commercial availability of CM in Italy and Europe.

Willingness to try (WTT) CM was measured with the statement “I would be willing to try cultured meat in the near future”, measured on a 7-point Likert scale (1 = not at all willing; 7 = very willing) already used in previous research [22,60]. For the empirical analysis, this variable was subsequently transformed into a dichotomous variable, with respondents scoring below the median value coded as 0 (low willingness) and those scoring above the median as 1 (high willingness) [22,66].

Perceptions of different attributes of CM (namely naturalness, safety, nutritional content, expected taste and price) were assessed using a previously validated scale [34,67], while perceptions of its potential sustainability benefits were measured with four statements adapted from de Koning et al. [69], which had already been applied in a similar study conducted in Italy [34].

In order to assess consumers’ openness to adopting innovative food products, ten items from the food neophobia scale (FNS) [70] were used. Similarly, to assess consumers’ reluctance to eat foods produced using novel technologies, five items selected from the food technophobia scale (FTNS) [71] were used, which have been validated in previous research [55,72]. As for socio-demographic information, sex at birth, age, area of residence, education, income, and dietary patterns were collected.

The questionnaire was designed to be completed within approximately 10 min. The measurement scales were translated into Italian while preserving the original meaning and conceptual equivalence of the items. To reduce the most common cognitive and methodological biases (e.g., order bias and primacy/recency effects), multiple randomization techniques were implemented. Furthermore, a pre-test with a pilot sample involving 30 students was conducted to verify the questionnaire’s comprehensibility and prevent wording bias. Based on pre-test results, minor adjustments were made.

### 3.2. Recruitment and Data Collection

The online survey administration took place between November and December 2024. The recruitment relied on voluntary self-selection. Participation was voluntary and completely anonymous. The survey was advertised through social media (i.e., Facebook and Instagram), via email (institutional mailing list), and by word of mouth. To prevent duplicate responses, an IP filter was applied.

Before starting the questionnaire, participants were informed that participation was voluntary, that no reward was offered, and that data would be collected anonymously and analyzed only in aggregate. They were also asked to provide informed consent. Participants were eligible if they met two inclusion criteria: (i) they were at least 18 years old, and (ii) they were enrolled in a university degree course. From an initial group of 1200 students who accessed the survey, 350 completed the survey; however, 15 were excluded from the analysis because they completed the questionnaire too quickly (significantly below the median time of the entire sample). The final sample therefore consisted of 335 valid observations.

### 3.3. Data Analysis

To characterize the sample, descriptive statistics were first calculated for all variables, including participants’ meat purchasing and consumption habits, their familiarity with CM, and their perceptions of the product.

To analyze the determinants of participants’ willingness to try CM, a binary logistic regression analysis was conducted. Logistic regression is a widely used statistical method in consumer behavior studies and has previously been applied in studies on the same topic [22,27,66]. Following Verbeke et al. (2021) [22], given the skewed distribution of the response, the dependent variable was analyzed as a discrete decision. Specifically, a dichotomous dependent variable was created by classifying respondents with a WTT score below the median as 0 (low willingness) and those with a score above the median as 1 (high willingness). This approach is consistent with previous research on novel and hypothetical food technologies, where consumer responses are often interpreted as reflecting an early adoption decision rather than as a continuous behavioral measure [22,66,73]. In the present study, treating WTT as a binary variable was considered appropriate because CM remains a largely hypothetical product that is not yet commercially available or widely consumed [22,27,73]. Moreover, this operationalization allows the analysis to focus on individuals who exhibit an early propensity to adopt the innovation, thereby distinguishing potential early adopters from consumers who remain skeptical or unwilling to try the product.

Moreover, dichotomization is consistent with the DOI framework, which theorizes adoption as a behavioral decision in which individuals move from non-adoption to adoption [29]. Accordingly, modeling WTT as a binary outcome facilitates the interpretation of the results in terms of the probability of acceptance among potential early adopters, an aspect that is particularly relevant in the context of emerging food technologies that have not yet entered the market.

The explanatory variables included in the model comprised measures of familiarity with CM, intention to change future meat-eating patterns, and perception of product attributes, as well as scores on relevant psychological scales. Socio-demographic variables such as gender and diet pattern were also considered as covariates. All predictors were entered simultaneously using the enter method. The model was estimated using maximum likelihood estimation. Model performance was evaluated with classification accuracy, pseudo-R^2^ (Cox & Snell and Nagelkerke), and goodness of fit diagnostics (Hosmer–Lemeshow test). Predictors were retained in the model based on theoretical relevance. All analyses were performed using IBM SPSS Statistics version 27 (SPSS Inc., Chicago, IL, USA).

## 4. Results

### 4.1. Characteristics of the Respondents

Table 1 summarizes the main characteristics of the respondents. The valid sample consisted of 335 students, of whom 51.5% were female, and 35% were aged between 20 and 22 years. In terms of education, 58% of respondents held a high school diploma, while 31% held a bachelor’s degree and 11% a master’s degree. The main fields of study were economics, business administration, and law, accounting for 78% of the sample. Most respondents lived in suburban areas (67%). With reference to income, 33% of respondents reported a family monthly income consistent with the national average (Italian National Institute of Statistics ISTAT, 2023) [74]. Finally, 63% of interviewees reported following an omnivorous diet, while 21% reported following a protein-rich diet.

With reference to meat consumption patterns and personal beliefs about meat consumption, the majority of respondents (63%) reported consuming fresh and processed meat two to four times per week. Most respondents considered animal meat consumption to be irreplaceable in their diet (43%) and believed that meat is necessary for maintaining a complete and balanced diet (41%). Furthermore, 37% of respondents were not willing to reduce their meat consumption in the near future. Among those expressing an intention to reduce meat consumption, the primary motivations were health concerns (25.6%), followed by concerns about animal welfare (23.5%). Regarding consumers’ concern for animal welfare, most respondents believe that it is important that the food they consume daily is produced without causing animals pain (29.2% completely agreed and 14.4% strongly agreed) and with respect for animal rights (41.3% completely agreed).

### 4.2. Respondents’ Familiarity and Perception of CM

Overall, respondents’ familiarity with alternative protein sources as substitutes for traditional meat was relatively high: 27% of respondents reported being very informed about this topic, while 19% reported being informed. However, with specific reference to CM, most respondents (52%) reported low familiarity. This lack of familiarity was also confirmed by the objective knowledge assessment, considering that in 60% of cases, respondents were unable to answer whether the production and consumption of CM have been authorized within the EU and Italy.

In terms of perception of product attributes, CM was perceived as more expensive than conventional meat (39% of respondents), less natural (51.5%), and less flavorful (32.5%). By contrast, its safety and nutritional properties were perceived as broadly comparable to those of conventional meat by 33% and 32% of cases, respectively.

Consumers’ perception of potential sustainability benefits derived from CM were generally positive. Specifically, 44.6% of respondents strongly agree with the statement that CM promotes animal welfare, 35% consider it climate-friendly, and 33% strongly agree with the statement that CM contributes to the preservation of natural resources. Greater uncertainty emerged regarding CM’s ability to contribute to alleviating hunger in developing countries, with more than 40% indicating that they neither agreed nor disagreed with this statement.

### 4.3. Food Neophobia (FNS) and Technophobia (FTNS)

As detailed in Table 2, respondents reported a moderate level of openness toward adopting innovative food products, as indicated by a mean value of the food neophobia scale (3.49). The highest mean scores were observed for the following items: “*I am very particular about the foods I eat*” (4.44), “*I like foods from different cultures*” (3.65), “*I am constantly sampling new and different foods*” (3.59), and “*At dinner parties, I will try new foods*” (3.44). With regard to novel technologies, the mean value of food technology neophobia was 3.67. Respondents seem quite confident that innovations in food technology can help people maintain a balanced diet (3.72); however, they also agreed that new food technologies diminish the natural quality of food (3.96).

To facilitate inclusion of FNS and FTNS in the logistic regression analysis, we used principal component analysis (PCA) with varimax rotation to reduce the number of variables and mitigate potential multicollinearity. The PCA was conducted for data reduction and exploratory purposes. Before conducting the principal component analysis, the suitability of the data for factor extraction was assessed using the Kaiser–Meyer–Olkin (KMO) measure and Bartlett’s test of sphericity. The FNS demonstrated good sampling adequacy (KMO = 0.808), whereas the FTNS showed acceptable adequacy (KMO = 0.645). Bartlett’s test was statistically significant for both scales (*p* < 0.001), confirming that the correlation matrix was appropriate for dimensionality reduction.

For both scales, a single component was extracted. Table 2 shows the mean score of each item of both FNS and FTNS, the factor loadings, and the percentage of variance explained by the extracted component. Both scales demonstrated adequate reliability, with Cronbach’s Alpha value above the cut-off level of 0.70.

### 4.4. Drivers of Willingness to Try (WTT)

Table 3 summarizes the results of the binary logistic regression analysis conducted to examine the drivers influencing respondents’ WTT for CM. The model was statistically significant (χ^2^ = 133.431, *p* < 0.001), indicating that the set of predictors reliably distinguished between respondents with high and low WTT. The model demonstrated good explanatory power, with pseudo-R^2^ values of 0.354 (Cox & Snell) and 0.495 (Nagelkerke), suggesting that approximately between 35% and 50% of the variance in the dependent variable was explained. Model fit was satisfactory, as indicated by the Hosmer–Lemeshow test (χ^2^ = 15.244, *p* = 0.055). The overall classification accuracy was 80.3%.

Several predictors were found to have a statistically significant positive effect on WTT for CM. Familiarity with CM was positively associated with the WTT (β = 0.371, *p* < 0.001), with an odds ratio (OR) of 1.450, indicating that greater familiarity increased the odds by approximately 45%. Perceived safety emerged as one of the strongest predictors (β = 0.686, *p* < 0.001; OR = 1.978), suggesting that higher perceived safety nearly doubled the likelihood of belonging to the high-WTT group. Similarly, perceived taste had a significant positive effect (β = 0.458, *p* = 0.011; OR = 1.581), indicating that more favorable taste perceptions substantially increased respondents’ willingness to try CM. Conversely, perceived naturalness, perceived nutritional value, and perceived price did not show statistically significant effects.

Ethical concerns regarding animal welfare also played a key role. Both general concern for animal welfare in daily food choices (β = 0.263, *p* = 0.007) and perceived animal welfare benefits from CM (β = 0.506, *p* = 0.001) were positively associated with WTT.

Neither food neophobia (FNS) nor food technology neophobia (FTNS) had a statistically significant effect on WTT. Likewise, respondents’ intention to reduce meat consumption in the future did not significantly predict willingness to try CM. Although some of these variables exhibited positive or negative coefficients, their effects were not statistically distinguishable from zero. Furthermore, demographic variables such as gender and education level were not significant predictors of the outcome.

## 5. Discussion

The present study contributes to the growing body of literature on university students’ acceptance and WTT of CM by examining both product-related attributes and personal value motivations through the lens of the DOI framework [29].

Overall, the descriptive analysis showed that university students in our sample had limited familiarity with and knowledge regarding CM. Furthermore, they perceived CM as being less natural and less flavorful than traditional meat, whereas the perception of potential sustainability benefits derived from CM was generally positive.

Interpreting the findings through the DOI framework, our results indicate that among *perceived benefit advantages*, ethical considerations, particularly those pertaining to animal welfare, play a significant role in promoting acceptance, both directly and through the perceived benefits of CM, which is consistent with previous evidence [30]. This finding aligns with the principles of moral motivation frameworks [51], suggesting that ethical values can serve as catalysts for early adoption. By contrast, the environmental (climate) benefits did not significantly predict WTT, corroborating previous research indicating that sustainability alone may not be sufficient to prompt behavioral change [43,65]. Similarly, expected taste emerged as a significant predictor of WTT, which is in line with past evidence [41,44]. Although many respondents indicated they thought CM would have less flavor than traditional meat, those reporting more favorable taste expectations were significantly more likely to express a willingness to try it. This provides additional support to the argument that sensory expectations can serve as a major barrier to behavior and reinforces the indication that rejection is often anticipatory and not experiential [61]. Interestingly, among product characteristics, perceived nutritional value and price were not significant predictors of WTT. A possible explanation is that CM remains largely a hypothetical product for most consumers, making evaluations of economic and nutritional attributes more speculative than experience-based. Moreover, the relatively homogeneous sample of university students may reduce variability in economic considerations.

With reference to *compatibility* with consumer values and habits, our results show that perceived naturalness did not significantly affect WTT, countering previous research [46,53]. At the same time, personal traits such as food neophobia and food technophobia did not significantly influence WTT. This finding contrasts with part of the literature [19,42,54] but is in line with those of Asioli et al. (2022) [63], who identified younger consumers characterized by a lower degree of neophobia toward new food technologies. This may reflect the specific characteristics of university students who generally exhibit greater openness to innovation and greater exposure to technological developments than older generations [32]. Nevertheless, the non-significant findings for FNS and FTNS should be interpreted with caution, given the low factor loadings observed for some items.

Concerning *complexity*, safety emerged as the most influential factor, confirming previous studies [23,34]. This result supports the evidence that, in the case of novel food technologies, risk perception remains a primary barrier to adoption [37]. Although respondents in our sample did not perceive CM as being significantly less safe than conventional meat, their evaluations of its safety strongly influenced their willingness to try it. Consequently, the provision of transparent information regarding the production process may serve to alleviate concerns pertaining to naturalness and safety, thereby enhancing support [32,51]. Hence, harmonized food safety and labeling standards will be essential once the product becomes available to ensure transparency and consumer trust, while balancing transparency with consumer comprehension [75].

Previous research showed that novel food technologies are often evaluated through a precautionary lens, especially when consumers lack direct experience with the product [60]. This reinforces the importance of transparent regulatory procedures and effective risk communication by public authorities, scientific institutions, and food safety agencies. Establishing trust in the regulatory process may therefore represent a critical prerequisite for market acceptance. Likewise, familiarity proved to be a very influential determinant of WTT, supporting the previous literature [21,61]. It is worth noting that while there were high levels of general awareness of alternative proteins in general, the objective knowledge of CM remained limited, suggesting that knowledge gaps still exist. This would suggest that increased exposure to information about CM as well as information about the production technology may reduce uncertainty and increase acceptance. In this regard, it is important to avoid the pitfalls experienced by genetically modified food companies, who have often been criticized for their lack of transparency, while also avoiding unintentionally framing of CM as unnatural or overly technological [76].

Even though *trialability and observability* were not assessed in the current study, the results indirectly suggest their relevance. Indeed, WTT appears to rely more on taste expectations rather than actual sensory evaluation, suggesting that future commercialization strategies offering actual tasting opportunities or other forms of direct experience may reduce uncertainty and prompt adoption [61].

With regard to communication, young consumers’ marked concern for animal welfare suggests that this aspect of CM should be emphasized. Overall, public campaigns aimed at increasing awareness of the environmental impact of conventional livestock farming may encourage a desirable reduction in meat consumption and foster the adoption of alternative protein sources, including CM [76].

Finally, our results show that socio-demographic variables were not significant predictors of WTT, supporting the mixed evidence reported in prior studies [52]. This reinforces the hypothesis that acceptance of CM among younger consumers is increasingly driven by personally perceived benefit rather than by traditional demographic characteristics. Accordingly, consumer segmentation strategies based on demographic variables may be less effective than approaches based on attitudes, knowledge, and value orientations.

## 6. Conclusions

Overall findings from current research, although specific to the investigated context, provide further evidence that early-stage acceptance of emerging food technologies, such as CM, is primarily shaped by *complexity factors* associated with uncertainty reduction mechanisms, such as familiarity and perceived safety, rather than by *compatibility* with personality traits. Among the *perceived benefit advantages*, ethical considerations related to animal welfare emerged as particularly influential.

The salience of perceived safety underscores the critical role of regulatory authorities as transparent and credible sources of information, which is essential for fostering public trust in the food-safety regulatory framework governing these products.

Our findings suggest that university students may constitute a primary target group for disseminating information about CM. Furthermore, messages emphasizing product safety and animal welfare appear to be particularly effective in increasing acceptance and willingness to try the product. These findings provide useful insights for policymakers and companies interested in promoting the market introduction of cultured meat among this specific consumer segment.

Despite its contributions, this study has several limitations that should be considered when interpreting the findings. First, the use of a convenience sample composed exclusively of university students limits the generalizability of the results to the broader Italian population. Young adults enrolled in higher education are typically more exposed to scientific information, technological innovations, and sustainability issues; therefore, the findings should be interpreted as referring to this specific population segment, which may represent potential early adopters. Furthermore, the recruitment strategy, based on voluntary participation through online channels, may have introduced self-selection bias, as individuals with a greater interest in food innovation or sustainability topics may have been more inclined to participate. Moreover, the absence of probability-based sampling prevents the estimation of sampling error and limits the external validity of the study. Additionally, the analysis is based on a hypothetical scenario as CM is not yet available in Italy. Therefore, stated willingness to try may not fully translate into actual behavior once the product becomes available on the market. Real-market experiments would be valuable to assess behavioral responses over time. Finally, the decision to dichotomize the WTT variable should also be considered when interpreting the findings, as this methodological choice inevitably entails some loss of information.

In addition, although several product-related perceptions and individual characteristics were included in the analysis, other potentially relevant determinants, such as trust in scientific institutions and regulatory authorities, were not investigated. Future research incorporating these variables could provide a more comprehensive understanding of the psychological mechanisms underlying consumer acceptance of CM.

## Figures and Tables

**Table 1:** Respondent’s characteristics (% of total respondents).

Sex at Birth	Female	51.5
	Male	45.5
	Prefer not to say	3
Age range	18–20	26.2
	20–22	34.8
	23–25	24.4
	26–28	10.5
	28–30	4.2
Residence area	Urban	33
	Sub-urban	67
Level of education	High school diploma	58.4
	Bachelor’s degree	31.1
	Master’s degree	10.5
Family monthly income (compared with national average)	Below	21.6
	Average	33.1
	High	14.5
	Prefer not to say	30.5
Dietary pattern	Vegan/vegetarian	5.8
	Protein-rich	21.2
	Gluten/lactose-free	4.3
	Omnivore	63
	Other	5.0
Meat consumption frequency (fresh and processed)	1 time per week	16
	2–4 times per week	63
	5–6 times a week	15
	Everyday	6
Intention to reduce meat consumption	Yes, for my health	25.6
	Yes, for animal welfare concerns	23.5
	Yes, for environmental concerns	13.6
	No	37.3

**Table 2:** Scale reliabilities and factor loadings.

	Mean Value	Standard Deviation	Factor Loadings	Cronbach’s Alpha	% Variance Explained
*Food Neophobia*	3.49			0.767	61.26%
I am constantly sampling new and different food *	3.59	1.880	0.838		
I don’t trust new foods	3.28	1.733	0.733		
If I don’t know what a food is I won’t try it.	3.52	1.904	0.735		
I like foods from different cultures *	3.65	1.929	0.826		
Ethnic food looks too weird to eat	3.18	1.821	0.724		
At dinner parties. I will try new foods *	3.44	1.850	0.878		
I am afraid to eat things I have never had before	2.98	1.776	0.815		
I am very particular about the foods I eat	4.44	1.829	0.636		
I will eat almost anything *	3.25	1.869	0.770		
I like to try new ethnic restaurants *	3.58	1.955	0.456		
*Food Technophobia*	3.67			0.732	56.87%
The benefits of new food technologies are often overestimated	3.94	1.594	0.796		
There are plenty of tasty foods available. So we don’t need to use new food technologies	3.45	1.615	0.862		
New food technologies diminish the natural quality of food	3.96	1.753	0.866		
New food technologies can help people maintain a balanced diet *	3.72.	1.585	0.428		
Innovations in food technology can help us produce food in a sustainable way *	3.26.	1.629	0.558		

* Reverse items recoded prior to the PCA; rotation methods: Varimax with Kaiser normalization.

**Table 3:** Results of logistic regression.

Variables	Coef.	Std. Err.	z-Value	*p*-Value	Odds Ratio	95% CI (OR)
Familiarity with CM	0.371 ***	0.104	3.56	<0.001	1.450 ***	1.182–1.778
Intention to reduce meat consumption	−0.209	0.136	−1.53	0.125	0.811	0.621–1.060
Perceived safety of CM	0.686 ***	0.180	3.82	<0.001	1.987 ***	1.397–2.825
Perceived naturality of CM	−0.015	0.195	−0.08	0.940	0.985	0.672–1.444
Perceived taste of CM	0.458 *	0.181	2.53	0.011	1.581 *	1.109–2.255
Perceived nutritional value of CM	0.133	0.189	0.70	0.481	1.142	0.789–1.655
Perceived price of CM	0.133	0.158	0.84	0.399	1.143	0.838–1.557
FNS	−0.123	0.179	−0.69	0.491	0.884	0.623–1.255
FTNS	−0.180	0.183	−0.98	0.326	0.835	0.583–1.196
Animal welfare concerns in daily food choices	0.263 **	0.097	2.72	0.007	1.301 **	1.076–1.573
Perceived animal welfare benefits from CM	0.506 ***	0.154	3.28	0.001	1.658 ***	1.225–2.244
Perceived climate benefits from CM	−0.182	0.148	−1.22	0.221	0.834	0.624–1.115
Gender (ref. male)	—	—	—	0.602	—	—
Education (ref. bachelor’s degree)	—	—	—	0.279	—	—
Constant	−9.513 ***	2.902	−3.28	0.001	—	—
Chi-square	133.431 *					
Pseudo R^2^ (Cox & Snell)	0.354					
Pseudo R^2^ (Nagelkerke)	0.495					
Hosmer–Lemeshow test	χ^2^ = 15.244	(*p* = 0.055)				
Overall classification accuracy	80.3%					

* *p* < 0.05; ** *p* < 0.01; *** *p* < 0.001.

## Data Availability

The raw data supporting the conclusions of this article will be made available by the authors on request.

## Abbreviations

The following abbreviations are used in this manuscript:

CM	Cultured meat
DOI	Diffusion of innovation
WTT	Willingness to try
FNS	Food neophobia scale
FNTS	Food technophobia scale
PCA	Principal component analysis
KMO	Kaiser–Meyer–Olkin
